# Magnetic resonance imaging in patients with cardiac implantable electronic devices: the RESONANCE Spanish registry

**DOI:** 10.1093/europace/euae277

**Published:** 2024-11-05

**Authors:** Francisco Ruiz Mateas, Marcos Antonio Pérez, Fernando García López, Susana González, Ignasi Anguera Camós, Gabriel Gusi Tragant, María Robledo Irrañitu, Ignacio Fernández Lozano, Juan Gabriel Martínez, Francisco Javier Alzueta Rodríguez

**Affiliations:** Cardiology Department, Hospital Universitario Costa del Sol, A-7, Km 187, 29603 Marbella, Málaga, Spain; Cardiology Department, Complexo Hospitalario Universitario de Ourense, Ourense, Spain; Cardiology Department, Hospital Arquitecto Marcide, Ferrol, Spain; Cardiology Department, Hospital Universitario Marqués de Valdecilla, Santander, Spain; Cardiology Department, Hospital Universitario de Bellvitge, Barcelona, Spain; Cardiology Department, Hospital Universitari Parc Taulí, Sabadell, Spain; Cardiology Department, Hospital Universitario de Araba, Vitoria-Gasteiz, Spain; Cardiology Department, Hospital Universitario Puerta de Hierro, Madrid, Spain; Cardiology Department, Hospital General Universitario Dr. Balmis, Instituto de Investigación Sanitaria y Biomédica de Alicante (ISABIAL), Alicante, Spain; Cardiology Department, Hospital Universitario Virgen de la Victoria, Campus de Teatinos, S/N, 29010 Malaga, Spain

**Keywords:** Magnetic resonance imaging, Cardiac implantable electronic devices, Pacemaker, Implantable cardioverter defibrillator

## Abstract

**Aims:**

Despite increasing evidence demonstrating the safety of magnetic resonance imaging (MRI) in patients with cardiac implantable electronic devices (CIEDs), this procedure is often neglected in this population. This Spanish registry aimed to determine the proportion of MRI referrals and performance among patients with pacemakers (PMs) or implantable cardioverter defibrillators (ICDs).

**Methods and results:**

This prospective, multicentre, open-label registry involved 21 Spanish centres. Data were collected upon implant of PMs or ICDs from BIOTRONIK and one year after, and included the number of MRIs and computed tomography scans prescribed, performed and denied, and reasons for denial. Data from 1105 patients (mean age: 74.2 years) were analysed and 982 completed the follow-up. Of them, 82.2% had a PM and 17.8% an ICD. A total of 351 imaging tests were prescribed in 220 patients (19.9%), including 52 MRIs in 39 patients (3.5%) and 299 computed tomography scans in 196 patients (17.8%). Among the MRIs, 44 (84.6%) were performed, five (9.6%) were not performed, and three (5.8%) were replaced by an alternative test. Most of the indicated computed tomography scans were performed (97.7%). The proportion of patients with an MRI scan referral was 4.6% during the pre-COVID-19 period and 2.6% during the COVID-19 period. No MRI-related arrhythmic ventricular event was reported.

**Conclusion:**

This registry revealed that only 3.5% of patients with CIEDs had an MRI referral over the study, with rates decreasing to 2.6% during the COVID-19 period. These rates contrast with the 85 MRIs conducted per 1000 inhabitants in Spain in 2020.

## Graphical Abstract

**Graphical Abstract:**
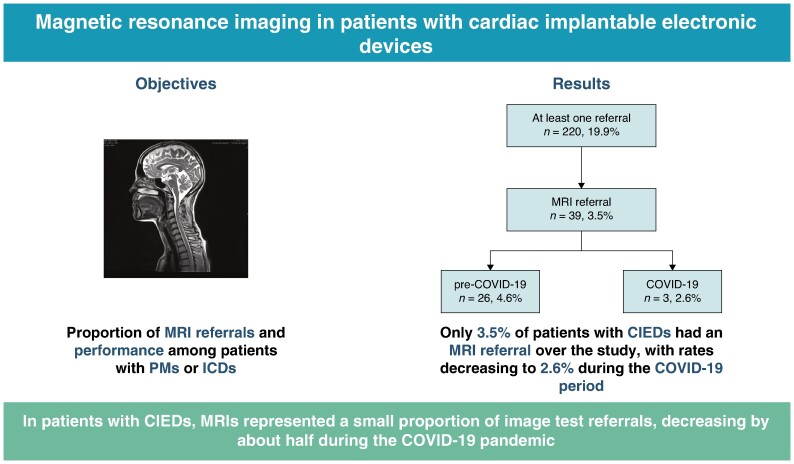

**What’s new?**

- In patients with cardiac implantable electronic devices, most imaging test referrals were computed tomography (CT) scans, while magnetic resonance imaging (MRI) represented a small proportion.
- Only 3.5% of patients with cardiac implantable electronic devices had an MRI referral, but most of these were carried out.
- The COVID-19 pandemic led to a substantial reduction in both MRI and CT scan referrals, with numbers decreasing by about half.

## Introduction

Magnetic resonance imaging (MRI) has become the gold standard diagnostic tool for an increasing range of indications, including cancer, neurological, musculoskeletal, and cardiac disorders.^1^ Key advantages of MRI are its excellent spatial resolution and the ability to identify soft tissues without ionizing radiation or iodinated contrast agents.^2^ As the use of MRI continues to grow, there is an increasing number of patients with cardiac implantable electronic devices (CIEDs), particularly among the elderly population, who often suffer from multiple comorbidities and require accurate diagnostic tools.^2^ In fact, it is estimated that up to 75% of patients with CIEDs will need an MRI scan at some point in their lifetime, and this rate is progressively rising.^3,4^ However, access to MRI for patients with CIEDs remains limited due to potential safety concerns.^5^ The interaction between CIEDs and MRI has historically been associated with potential hazards caused by static, gradient, and radiofrequency fields, which could lead to device malfunction and harm the patient.^6^ To minimize these complications, devices and leads have been modified, resulting in the development of MRI-conditional CIEDs, significantly reducing the incidence of MRI-related complications.^7,8^

Several studies demonstrated the safety of MRI in patients with both MRI-conditional^6,9–14^ and non-MRI-conditional devices^15,16^ when strict protocols are followed. Therefore, according to the latest guidelines from the European Society of Cardiology on cardiac pacing and cardiac resynchronization therapy, MRIs can be performed safely in patients with MRI-conditional pacemakers (PMs) when manufacturer’s instructions are followed (class IA) and if no alternative imaging modality is available in patients with non-MRI-conditional PMs (class IIa).^17^ The European Heart Rhythm Association (EHRA) consensus on prevention and management of interference due to medical procedures in patients with CIEDs recommends following a standardized protocol and checklist to treat CIED patients during MRI scans.^18^ A recent Spanish consensus also outlined a structured workflow for patients with CIEDs who require an MRI.^19^ The recommended workflow involves an initial device interrogation to assess pacing dependency and programming settings, followed by a subsequent interrogation to confirm proper functioning and enable any required reprogramming.^19^

Despite these recommendations, the performance of an MRI in the presence of a CIED is not yet streamlined since it requires training of medical personnel and coordination between departments. Accordingly, MRI scans are often denied to patients with CIEDs, even to those with MRI-conditional systems.^5^

To address this issue and enable safe MRI access for patients with CIEDs, it is crucial to ascertain the proportion of patients with CIEDs who undergo an MRI scan relative to those who are prescribed this imaging test. The aim of the RESONANCE registry was to assess the rates of prescription and performance of MRI examinations among patients with PMs and implantable cardioverter defibrillators (ICDs) in Spain.

## Methods

### Study design

The RESONANCE study was a prospective, multicentre, open-label registry conducted at 21 sites in Spain. The study adhered to the ethical principles of the Declaration of Helsinki and received approval from the Ethics Committee of Hospital Virgen de la Victoria (Málaga, Spain). All participants provided written informed consent.

Data were collected at baseline (within 30 days after device implant) and after one year (±30 days). Baseline data included demographic and clinical information such as underlying heart disease, history of atrial fibrillation, and left ventricular ejection fraction (LVEF). Additionally, device type, model, and lead placement were recorded, and the device was interrogated to verify appropriate electrical lead parameters and proper device function. Patients were provided with a patient card indicating whether the implanted system was MRI-conditional, along with a questionnaire to be completed in case an MRI or computed tomography (CT) scan was indicated. At the one-year follow-up visit, MRI-related adverse events were registered, and the device was interrogated. Depending on the activation of the remote monitoring feature, the follow-up visit could be conducted either on-site or remotely.

MRIs or CT scans were performed as per the routine clinical practice of participating centres. The following data were collected for MRI examinations prescribed during the follow-up period: the reasons for MRI denial, the time in asynchronous mode, the healthcare professional responsible for the referral or the denial, and any arrhythmic ventricular events observed during the procedure.

### Study population

Patients aged >18 years with PMs or ICDs and able to understand the nature and procedures of the study were included. Patients were excluded when had abandoned leads, epicardial leads, life expectancy < 12 months, cardiac transplant within the previous 6 months or expected within the following 3 months, cardiac surgery within the previous 3 months or planned within the following 3 months, irreversible brain injury, or were pregnant or breastfeeding.

### Study devices

Implanted CIEDs comprised six different systems from BIOTRONIK (ICDs: Ilesto, Iforia, Ilivia; PMs: Evia, Philos II, and Edora) equipped with the Home Monitoring feature. Devices were used following manufacturer’s recommendations. All devices were labelled MRI-conditional when used in combination with the respectively labelled leads.

The Ilivia and Edora systems are equipped with the MRI AutoDetect, a feature to automatically detect MRI fields, switching the device into a safe mode when the patient enters the field and returning to permanent program upon exiting the field.^20^ For systems without the MRI AutoDetect function, the asynchronous mode was manually programmed.

### Study outcomes

The main objective of the study was to determine the proportion of MRI referrals and performance among patients with PMs or ICDs over one year of follow-up. To this end, the primary endpoint was the proportion of MRIs prescribed, performed and denied.

Secondary endpoints were (i) the proportion of MRIs replaced by alternative imaging tests among those denied, (ii) the reasons for MRI scan denial, (iii) the proportion of CT scans prescribed, performed and denied, (iv) the mean time in asynchronous mode during the MRI, (v) the proportion of patients with the MRI AutoDetect activated and the efficacy and safety of this feature, (vi) the healthcare professional responsible for the MRI referral, and (vii) the number of arrhythmic ventricular events during the MRI.

### Statistical analysis and sample size

While previous estimations suggested that 50−75% of patients would require an MRI examination over the lifetime of their device,^21^ these percentages may not be directly applicable to Europe or Spain. In worst-case scenario, the parameter to be measured for the primary efficacy variable was assumed to be 0.5. Considering a confidence level of 95%, an error of 3% and a dropout rate of 20%, the estimated number of patients to be included was 1248.

To examine the patient population with imaging test referrals, data were analysed during the whole study period and distinguishing between the periods before the onset and during the COVID-19 pandemic. For this purpose, the pre-COVID-19 period included patients enrolled from May 2017 to January 2019 and followed for one year, and the COVID-19 period encompassed patients enrolled from January 2020 to May 2020 and followed for one year. Patients enrolled between January 2019 and January 2020 were excluded from that specific analysis to avoid heterogenous circumstances during the follow-up period.

Statistical analyses were performed using the SAS software (SAS Institute, Cary, SC, USA) for Windows, version 9.4. Continuous variables were described using measures of central tendency such as mean, standard deviation, median, and interquartile range. Categorical variables were presented by numbers and percentages.

## Results

### Patient and device characteristics

**Figure 1:**
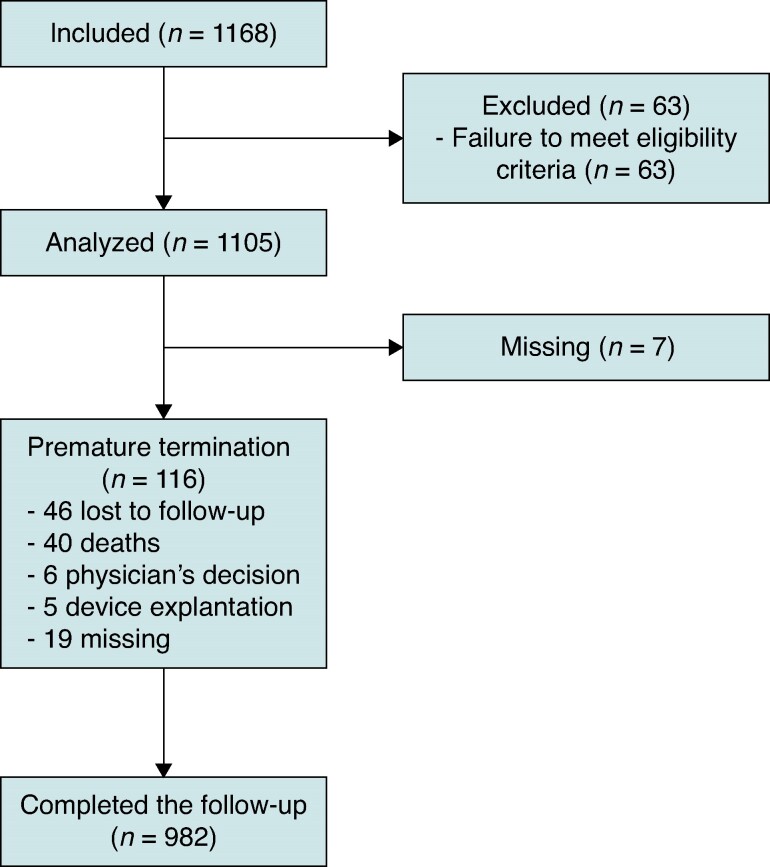
Flow chart showing the study design.

From May 2017 to May 2020, 1168 patients from 21 Spanish centres were included in the registry. Data from 1105 patients were analysed, and from 63 were excluded because they did not meet the selection criteria. A total of 982 patients completed the one-year follow-up, and 116 prematurely terminated. Of them, 46 were lost to follow-up, 40 died during the study, six terminated because of physician’s decision, and five had the device explanted (*Figure 1*). The reasons for the six terminated follow-ups because of physician’s decision were: PM pocket’s complications, end-stage cancer, elective transplant, heart transplant, device upgrade, and upgrade to CRT. Subject disposition across the different time periods was as follows: 567 subjects (51.3%) were included and completed the follow-up in the pre-COVID-19 period and 117 (10.6%) during the COVID-19 pandemic. The remaining 421 patients (38.1%) were included between both periods.

Mean age in the overall population was 74.2 years. At baseline, 235 (21.3%) patients had ischemic cardiomyopathy, 143 (13.0%) valvular heart disease, 285 (25.8%) heart failure, and 398 (36.2%) atrial fibrillation (thereof 49.7% permanent, 38.7% paroxysmal, and 11.6% persistent). Mean baseline LVEF was 52.1%. Baseline characteristics are shown in *Table 1*.

**Table 1:** Clinicaland demographic characteristics of study participants

Variables	Total	Value
Age (years), mean ± SD	1105	74.2 ± 11.2
Median (IQR)		76.0 (67.0−83.0)
NYHA class	1052	
I		241 (22.9%)
II		254 (24.1%)
II–III		55 (5.2%)
III		9 (0.9%)
No heart failure		493 (46.9%)
Cardiomyopathy	456	
Ischaemic		235 (51.5%)
Non-ischaemic		159 (34.9%)
Other		62 (13.6%)
Valvular heart disease	1102	143 (13.0%)
Heart failure	1103	285 (25.8%)
History of AF	1100	
None		702 (63.8%)
Permanent		198 (18.0%)
Paroxysmal		154 (14.0%)
Persistent		46 (4.2%)
Previous AF ablation	1067	22 (2.1%)
History of thrombo-embolic events or stroke	1069	
None		993 (92.9%)
Stroke		58 (5.4%)
Transient ischaemic attack		15 (1.4%)
Peripheral arterial embolus		3 (0.3%)
LVEF (%), mean ± SD	596	52.1 ± 15.0
Median (IQR)		55.0 (40.0−64.0)

Data are expressed as *n* (%) unless otherwise specified.

AF, atrial fibrillation; IQR, interquartile range; LVEF, left ventricular ejection fraction; NYHA, New York Heart Association; SD, standard deviation.

Most patients had an implanted PM (907, 82.2%) and 197 (17.8%) an ICD. The most common systems were dual-chamber PMs (538, 59.7%), followed by single-chamber PMs (351, 39.0%) and single-chamber ICDs (107, 54.6%) (*Table 2*). Right ventricular lead placement was apical in 951 (88.0%) patients and septal in 130 (12.0%) patients. Within one month after device implant, 540 (49.2%) patients had the Home Monitoring activated (191 patients with ICDs and 349 with PMs).

**Table 2:** Main characteristics of the devices included

Implanted device	1104	*n* (%)
Pacemaker		907 (82.2%)
System		
Single chamber		351 (39.0%)
Dual chamber		538 (59.7%)
CRT-P		12 (1.4%)
*N* missing		6
Implantable cardioverter defibrillator		197 (17.8%)
System		
Single chamber		107 (54.6%)
Single chamber with atrial sensing (DX)		42 (21.4%)
Dual chamber		22 (11.2%)
CRT-D		25 (12.7%)
*N* missing		1

Data are expressed as *n* (%).

CRT, cardiac resynchronization therapy; ICD, implantable cardioverter defibrillator.

### MRI examinations

**Figure 2:**
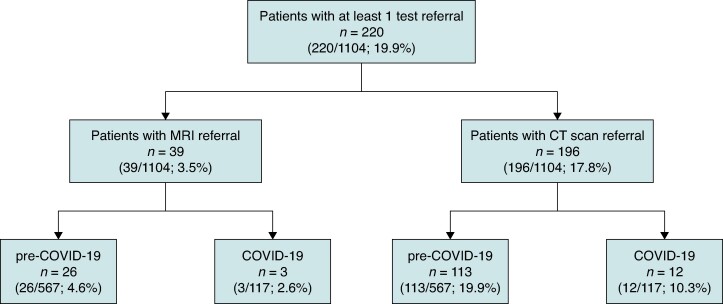
Flow chart showing the number of patients with imaging test referrals. Ten patients had an MRI and 71 a CT scan referral between January 2019 and January 2020 and were excluded from the COVID period analysis. MRI, magnetic resonance imaging; CT, computed tomography.

A total of 220 patients (19.9%) had at least one imaging test indicated (39 patients an MRI and 196 a CT scan), of whom 211 (95.9%) had the examination performed (33 patients an MRI and 192 a CT scan). Among the 567 patients enrolled during the pre-COVID-19 period, 130 (22.9%) were prescribed an imaging test, whereas only 14 (12.0%) of those included during the COVID-19 period (*n* = 117) had an imaging test referral. Specifically, during the pre-COVID-19 period, 4.6% out of 567 patients had an MRI scan referral and 19.9% a CT scan referral, whereas in the COVID-19 period, the percentages were lower, with 2.6% and 10.3% out of 117 patients having MRI and CT scan referrals, respectively (*Figure 2*).

**Figure 3:**
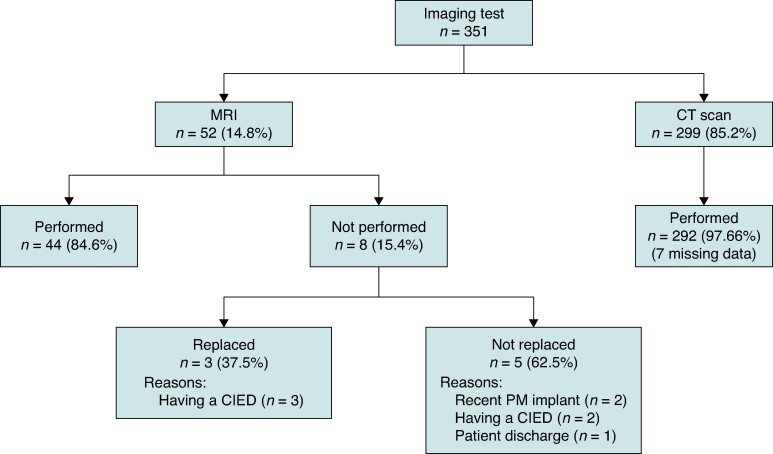
Flow chart showing imaging test performance in the study population. MRI, magnetic resonance imaging; CT, computed tomography; CIED, cardiac implantable electronic device; PM, pacemaker.

The number of imaging test referrals was 351, 52 (14.8%) MRIs and 299 (85.2%) CT scans. Out of the 52 MRIs requested, 44 (84.6%) were performed and eight (15.4%) were not performed. Of those not performed, three (37.5%) were replaced by an alternative test (all of them because of having a CIED), and five (62.5%) were not replaced (two because of PM implant within <2 weeks, two because of having a CIED, and one because the patient was discharged). All MRIs were replaced by a CT scan. Regarding CT scans, 292 (97.7%) were performed, and the remaining 7 were missing data (*Figure 3*).

MRIs were mainly prescribed by internal medicine specialists (10/52, 19.2%), followed by neurologists (8/52, 15.4%), oncologists (8/52, 15.4%), and traumatologists (8/52, 15.4%). CT scans were mainly prescribed by emergency medicine physicians (76/298, 25.5%), followed by cardiologists (28/298, 9.4%), and oncologists (24/298, 8.1%). The widest differences between prescribed MRIs and CT scans were observed in the following specialties: emergency department (2.0% vs. 25.5%), traumatology (15.7% vs. 3.7%), and neurology (15.7% vs. 7.4%).

No MRI-related arrhythmic ventricular event was registered. The mean time in asynchronous mode during the MRI was 1.2 h, and the MRI AutoDetect feature was activated in 14 out of 42 patients (33.3%). Physicians considered that this feature allowed to reduce the time needed per patient and reported that it automatically switched back to permanent program in patients with available data for these variables (*n* = 12).

## Discussion

Although having a CIED is no longer an absolute contraindication for accessing MRI, patients with implanted devices are less likely to be referred than those without devices.^5^ MRI is often substituted by less suitable imaging techniques, despite increasing evidence demonstrating the safety of MRI in this population when manufacturer’s guidance is followed and the availability of protocols to minimize risks during the process.^17,22,23^ Therefore, it is crucial to determine the proportion of patients with CIEDs undergoing MRI to evaluate adherence to current recommendations. This is the first Spanish registry describing the rates of MRI prescription and performance among patients with CIEDs.

We analysed data from 1105 patients, comprising one of the largest cohorts in this population. Our cohort included both patients with PMs and ICDs, although most patients had a PM (81%), contrasting with an Italian registry in which patients with PMs accounted for 49% of the population.^10^

An important finding from our registry is that only 3.5% of patients with CIEDs had an MRI referral. This rate seems lower than expected for a population with an average age of 74 years and comorbidities. Indeed, data from the Organization for Economic Co-operation and Development indicate that Spain performed 85 MRIs per 1000 inhabitants in 2020, with an increasing trend per year.^24^ Therefore, although we cannot be certain, we speculate that patients with CIEDs may be under-referred probably due to a lack of awareness regarding device compatibility and insufficient coordination between services. This lower rate of referrals in patients with CIEDs aligns with a previous study showing significantly fewer MRI examinations in patients with ICDs compared to those without implanted devices.^5^ The proportion of patients with MRI indications is lower than that observed in an Italian registry (6.9%).^10^ Notably, results before the onset of the COVID-19 pandemic are closer to this figure, whereas a substantial decrease in imaging test referrals was observed during this period. This suggests that under-referral may not be unique to Spain, but rather a more widespread issue. Another factor to highlight is that the Italian registry reported a higher rate of MRI referrals among patients with PMs, which represented 49% of their cohort^10%^ vs. 81% in our study. Still, the 3.5% rate of MRI referrals in our study seems low, considering that most patients had MRI-conditional devices. In this regard, the Italian registry observed that having a complete MRI-conditional system was not a significant determinant of MRI Access.^10^ Moreover, a survey conducted in all hospitals with MRI in England revealed that only 46% offered MRI scans to patients with CIEDs, and 85% of the departments performed less than 10 scans annually.^25^ The most commonly cited barriers were safety concerns, logistical difficulties, and the lack of cardiology support.^25^ In this context, it would be interesting to analyse the main obstacles to prescribing MRI in patients with CIEDs in the Spanish setting. Likely, the lack of protocols and coordination between departments influenced the number of MRI referrals in our cohort. Another important determinant in our data was the impact of the COVID-19 pandemic, leading to approximately a 50% reduction in the number of MRI and CT referrals. In Spain, as in many other countries, a large number of therapeutic and imaging procedures were temporarily halted as part of the response to the health crisis. Previous European studies found a negative impact of the pandemic on the workload of radiology services^26^ and on the care of patients with cardiac diseases.^27^

Interestingly, most of the MRI examinations prescribed were performed, although a non-neglected 15% proportion of patients received an alternative test or no test at all. This rate is substantially lower than the 38% of denied MRIs reported in the Italian registry.^10^ Again, the study found significant differences in the proportion of MRI examinations denied between patients with PMs and ICDs, with a higher proportion in the latter group.^10^ Although the rate of MRI scans performed was high in our study, it is important to highlight the consequences of delaying or substituting this unmatched technique for patients. A prospective study found that MRI added value to patient diagnosis in 97% of cases and changed the primary diagnosis in 30% of patients.^28^

Taken together, our results reveal that the low rate of MRIs performed is mainly due to a scarce number of referrals in this population. Specifically, internal medicine specialists, neurologists, oncologists, and traumatologists were the primary prescribers of MRIs. Compared to CT scans, the emergency department and pneumology were the specialties with the lowest MRI referrals. Additionally, all MRIs were denied by radiologists, which is consistent with previous observations.^10^ This reinforces the importance of establishing and implementing protocols that facilitate coordination between services and streamline the workflow.^8^ Indeed, the EHRA consensus recommends performing MRIs in patients with CIEDs in centres with appropriate teams, protocols, and equipment and establishing collaborative relationships between specialists to ensure safe outcomes.^18^ One of the improvements aimed at simplifying the workflow is the MRI AutoDetect, which was activated in 14 cases (33.3%) in our cohort of patients that underwent MRI. A retrospective study of MRI-conditional systems in the USA found that this feature was activated in 71.3% of systems.^20^

Although our registry was not specifically designed for this purpose, no MRI-related arrhythmic ventricular event was detected during the procedure. This low rate of complications aligns with clinical studies assessing the safety of BIOTRONIK MRI-conditional devices under specific MRI conditions.^11–14^ Although MRIs scans were conducted at 1.5T in these studies, a retrospective study including devices from BIOTRONIK found that 3T MRI scans were safe in patients with ‘3T MRI-conditional’ and ‘non-3T MRI-conditional’ CIEDs.^29^

Our study has some limitations. First, despite being one of the largest cohorts of patients with CIEDs, the limited number of MRI referrals prevented us from comparing data between patients with PMs and ICDs, especially considering that patients with ICDs only represented 18% of the population. Secondly, it is also likely that the number of MRIs performed was insufficient to detect rare adverse events associated with the procedure. Lastly, results may not be generalizable to devices from other manufacturers different from BIOTRONIK and are limited by the study design, which excluded certain patient subsets, such as those with abandoned and epicardial leads, which are included in specific studies.^30,31^

Despite these limitations, our nationwide registry provides insight into MRI access among patients with CIEDs in Spain based on a large cohort. The data reveal an under-referral of MRI in patients with CIEDs, despite MRI-conditional certification. This low rate of MRI referrals was particularly evident during the COVID-19 pandemic, which also resulted in a decrease in the number of prescribed CT scans. These results underscore the importance of implementing guidelines and protocols to facilitate coordination between services and streamline the procedure.

## Data Availability

Data are available upon reasonable request.
